# Mutations in the epidermal growth factor receptor (EGFR) gene in triple negative breast cancer: possible implications for targeted therapy

**DOI:** 10.1186/bcr2857

**Published:** 2011-04-01

**Authors:** Yvonne Hui-Fang Teng, Wai-Jin Tan, Aye-Aye Thike, Poh-Yian Cheok, Gary Man-Kit Tse, Nan-Soon Wong, George Wai-Cheong Yip, Boon-Huat Bay, Puay-Hoon Tan

**Affiliations:** 1 Department of Pathology, Singapore General Hospital, Outram Road, Singapore 169608, Singapore; 2 Department of Anatomical and Cellular Pathology, Chinese University of Hong Kong, Ngan Shing Street, Shatin, NT, SAR, PR China; 3 Department of Medical Oncology, National Cancer Centre Singapore, 11 Hospital Drive, Singapore 169610, Singapore; 4 Department of Anatomy, Yong Loo Lin School of Medicine, National University of Singapore, MD10, 4 Medical Drive, Singapore 117597, Singapore

## Abstract

**Introduction:**

Triple negative breast cancer is associated with poorer prognosis and unresponsiveness to endocrine and anti-HER2 directed agents. Despite emerging data supporting the use of polyADP-ribose polymerase (PARP) inhibitors, complete and durable responses are rare and exploration of additional targeted therapies is needed. Epidermal growth factor receptor (EGFR) is expressed in triple negative breast cancer and several clinical trials are testing the role of anti-EGFR directed therapy. However, the rate of EGFR mutations is poorly defined. We, therefore, sought to characterize EGFR mutations in triple negative breast cancers.

**Methods:**

Seventy samples were randomly chosen from a cohort of 653 triple negative breast tumours for EGFR mutation analysis. These samples were immunostained for EGFR protein expression and consisted of negatively stained and positively stained cases. DNA was extracted from paraffin blocks and polymerase chain reaction was performed to amplify exon regions 18 to 21 of the EGFR gene. Direct sequencing of the purified PCR products was performed.

**Results:**

EGFR mutations were found in 8 of 70 samples (11.4%). Mutations were predominantly exon 19 deletions (4 of 70 samples, 5.7%), which clustered in the region spanning codons 746 to 759 within the kinase domain of EGFR. Two types of exon 19 deletions were seen: a 15 nucleotide deletion (del E746-A750) (2 of 70 samples) and a 24 nucleotide deletion (del S752 - I759) (2 of 70 samples). Other exon 19 mutations observed were the inversion of the complementary strand (1 of 70 samples). Exon 21 mutations included missense substitution, L858R (1 of 70 samples) and T847I (2 of 70 samples). Mutations observed were independent of EGFR protein expression determined by immunohistochemical staining.

**Conclusions:**

This study is among the first to document the presence and estimate the prevalence of EGFR mutations in triple negative breast cancer. These findings have potential implications for the design of clinical trials involving anti-EGFR directed therapy which currently do not select for patients based on presence of activating EGFR mutations, which may hence be underpowered to detect significant benefit in unselected populations. More complete sampling of EGFR mutation status in triple negative breast cancer is needed to determine the true mutation rate.

## Introduction

Triple negative breast cancers, defined by the lack of estrogen receptor (ER), progesterone receptor (PR) and epidermal growth factor receptor 2 (Her2/cerbB2/EGFR2) expression, account for 10 to 20% of all breast carcinomas in Asian and Western populations [1-7], but occur at much higher frequencies in individuals of African descent [1-3,8]. These tumours are usually of higher histological grade (Grade 3) [1,3,4,6,9,10] and are associated with distinctive metastatic patterns [9,11], shorter time to recurrence and earlier mortality [9,11,12]. Recent focus on this breast cancer subtype relates to resistance to endocrine and anti-HER2 directed therapy, phenotypic similarity to breast cancers in BRCA1/2 mutation carriers and the development of polyADP-ribose polymerase (PARP) inhibitors which have demonstrated promising activity in this disease. Despite this breakthrough, sustained complete remissions in advanced triple negative breast cancer are rare and additional therapies directed against appropriate molecular targets are needed.

EGFR is a receptor tyrosine kinase important in transducing extracellular signals from the cell surface to the cell interior, mediating crucial processes such as cell proliferation, differentiation, migration and apoptosis. Dysregulated expression of these receptors can lead to aberration of homeostatic cellular processes, resulting in malignant transformation of cells. Activating EGFR mutations have been reported in cancers such as non-small cell lung cancer (NSCLC) and head and neck cancers and are predictive of response to gefitinib or erlotinib therapy [13-15]. EGFR protein is expressed in 30% to 52% of triple negative breast cancers [7,16,17] and up to 60% of the closely related basal-like breast cancers and is associated with poor prognosis [18-21]. These observations are the basis for a number of ongoing clinical trials which are exploring the role of monoclonal antibodies against EGFR such as cetuximab and EGFR tyrosine kinase inhibitors such as erlotinib in triple negative breast cancer.

Many mutations in the EGFR gene have been reported in NSCLC but only a few have been validated, either from *in vitro *studies or from tumour responses in NSCLC patients, to be associated with responses to EGFR tyrosine kinase drugs [13,14]. These mutations are usually found in exons 18, 19, 20 and 21, and include missense substitutions such as G719A/S and L858R and deletions like E746 to A750 (removal of amino acids Glucine-Leucine-Arginine-Glucine-Alanine (ELREA)) which are associated with sensitivity to tyrosine kinase inhibitors [13,14]. Mutations associated with resistance to EGFR tyrosine kinase inhibitors are D761Y [22] and T790M [23,24]. We sought to determine whether such mutations exist in triple negative breast cancers, the results of which may help to select patients suitable for inclusion in clinical trials evaluating the role of anti-EGFR directed therapies in this condition.

In this study, we report that 8 of 70 samples (11.4%) of triple negative breast cancers harbor EGFR mutations, including exon 19 deletions, inversions and exon 21 missense substitutions, which may predict sensitivity to EGFR tyrosine kinase drugs, thus suggesting a rationale for the clinical applicability of detecting EGFR mutations in these tumours, and potential use of EGFR tyrosine kinase inhibitor therapy.

## Materials and methods

### Histopathology and immunohistochemical review

Seventy archival paraffinised samples of triple negative breast cancer, derived from a cohort of 653 invasive triple negative breast tumours diagnosed at the Department of Pathology, Singapore General Hospital, were chosen for genomic EGFR mutation analysis. Triple negative status of these tumours was confirmed by negative ER, PR, and cerbB2 immunostaining. Briefly, paraffin sections were stained for ER using Neomarker RM9101-S (1:50 dilution), PR using Neomarker RM9102-S (1:200 dilution) and cerbB2 using Neomarker RM9103-S (1:200 dilution). Antigen retrieval was performed by heating in 0.01 M Tris EDTA pH9 using a microwave (Milestone T/T mega). The detection system used was Dako Envision Detection kit (K5007). For the purposes of this study, ER and PR staining was considered positive when at least 10% of tumour cells displayed a minimum of 2+ nuclear staining while cerbB2 was considered positive if at least 30% of tumour cells showed 3+ cell membrane staining. A borderline/equivocal result was given for cerbB2 when at least 10% of tumour cells demonstrated 2+ cytoplasmic membrane staining. Tumours that failed to fulfill any of the above criteria were considered triple negative.

Anti-EGFR monoclonal clone E30 (M7239, Dako, Glostrup, Denmark) was used at a dilution of 1:50. EGFR cytoplasmic membrane positivity was considered positive EGFR staining. Staining intensity was scored as 0 (no staining), 1+ (weak), 2+ (moderate) and 3+ (strong). The immunohistochemical staining profile of the 653 invasive triple negative breast tumours from which 70 breast tumour tissues used for EGFR mutational analysis were randomly selected from, is shown in Table 1. Ethics approval had been obtained from the Institutional Review Board, Singapore General Hospital. Samples were anonymised with waiver of informed consent.

**Table 1:** Immunohistochemical profile of EGFR IHC staining in 653 triple negative breast tumours

EGFR IHC staining	Number of cases (Percentage)
Negative (0)	455 (70%)
Weak (1+)	159 (24%)
Moderate (2+)	24 (4%)
Strong (3+)	15 (2%)
Total	653 (100%)

IHC, immunohistochemical.

### Mutation analysis

Representative unstained, formalin-fixed and paraffin-embedded (FFPE) tumour sections were macro-dissected to ensure purity of at least 70% invasive carcinoma cells and without the presence of any normal breast epithelial cells. Genomic DNA was extracted using QIAamp DNA extraction kit for FFPE tissues (Qiagen, Hilden, Germany) according to the manufacturer's protocol but with slight modifications. Briefly, paraffin was removed using xylene and residual xylene removed with ethanol. Buffer ATL was added to the deparaffinised tissue and heated at 98°C for 15 minutes [25,26] and cooled to room temperature. Proteinase K was then added to the tissue and incubated at 56°C for 16 hours. Afterward, the tissue mixture was incubated at 90°C for one hour and cooled to room temperature. Buffer AL was then added and the following wash steps were performed according to manufacturer's instructions. DNA yield and purity was quantitated and assessed using the Nanodrop (Thermo Fisher Scientific, Waltham, MA, USA).

Polymerase chain reaction (PCR) was then performed for all DNA samples using primers designed to amplify exons 18, 19, 20 and 21 of the EGFR gene. Primer sequences are shown in Table 2. Amplification reactions were set up using reagents included in the Taq PCR Core Kit (Qiagen), in accordance with the manufacturer's protocol. Essentially, each PCR reaction consisted of 1× PCR buffer, 0.2 mM dNTP, 0.3 uM forward and reverse primers, 1.25 U Taq DNA polymerase and 250 ng of genomic DNA in a total volume of 50 ul. The PCR cycling program was as follows: (1) 94°C for 4 minutes (1 cycle), (2) 94°C for 1 minute, 60°C for 1 minute, 72°C for 1 minute (40 cycles) and (3) 72°C for 10 minutes (1 cycle). Non-template (DNA) control represented the negative control and was included in every PCR run.

**Table 2:** Primer sequences

Exon	Forward Primer	Reverse Primer	PCR product size (bp)
Exon 18	CTGAGGTGACCCTTGTCTCTGTGTTCTT	AGAGGCCTGTGCCAGGGACCTTA	186
Exon 19 [24]	TCACTGGGCAGCATGTGGCA	CAGCTGCCAGACATGAGAAA	241
Exon 20	CCATGCGAAGCCACACTGA	CGTATCTCCCTTCCCTGATTACC	248
Exon 21	AGCAGGGTCTTCTCTGTTTCA	TGACCTAAAGCCACCTCCTT	200

bp, base pairs; PCR, polymerase chain reaction.

PCR products were analysed by performing electrophoresis on a 2% agarose gel stained with ethidium bromide. PCR products were purified using Qiagen PCR Purification Kit (Qiagen). The purified PCR amplicons were sequenced by 1^st ^BASE Pte Ltd (Singapore). Sequencing was carried out in both forward and reverse directions. DNA sequences were analysed using the National Center for Biotechnology Information Human EGFR gene sequence (mRNA Reference sequence NM_005228) and BLAST software. EGFR mutations detected in the initial round of sequencing were confirmed by subsequent rounds of independent polymerase chain reaction and sequencing reactions. Only mutations confirmed by subsequent rounds are reported. Cases found to harbor EGFR mutations were checked against the corresponding normal benign breast tissue consisting of at least 50% of epithelial cells to determine if mutations were somatic or germline.

## Results

### Tumour characteristics

Clinicopathological data for the 70 cases of triple negative breast cancers are shown in Table 3. The median patient age was 57 (range 38 to 78 years) and the patient cohort was predominantly Chinese (59 of 70 cases) with nine patients being of Malay and two patients of Indian ethnicity. Cases were predominantly invasive ductal carcinomas (*n *= 66), with two cases of invasive lobular carcinoma, one case of papillary carcinoma and one metaplastic carcinoma. The majority (53) samples were of histological grade 3 with 15 cases of grade 2, and two cases which were grade 1. Sixteen cases showed trabecular growth patterns while the remaining five cases demonstrated syncytial growth. The median tumour size was 34.9 mm (range 1.5 mm to 125 mm), 28 cases were associated with lymphovascular invasion, and 34 patients had axillary lymph node involvement.

**Table 3:** Clinicopathological characteristics of triple negative breast cancers studied in this cohort

Clinicopathological features	Numbers
**Age (years)**	
Mean	52
Minimum	38
Maximum	78
	
**Tumour size (mm)**	
Mean	34.9 mm
Minimum	1.5 mm
Maximum	125 mm
	
**Tumour grade**	
1	2
2	15
3	53
	
**Histology**	
Invasive ductal carcinoma	66
Invasive lobular carcinoma	2
Papillary	1
Metaplastic	1
	
**Growth pattern**	
Trabecular	38
Syncytial	32
	
**Nuclear pleomorphism**	
Moderate	19
Marked	51
	
**Lymphovascular invasion**	
Present	28
Absent	41
Not available	1
	
**Axillary lymph node involvement**	
Involved	34
Not involved	31
Not available	5
	
**Associated DCIS**	
Absent	19
Intermediate nuclear grade	28
High nuclear grade	11
Not available	12

DCIS, ductal carcinoma *in situ*.

**Figure 1:**
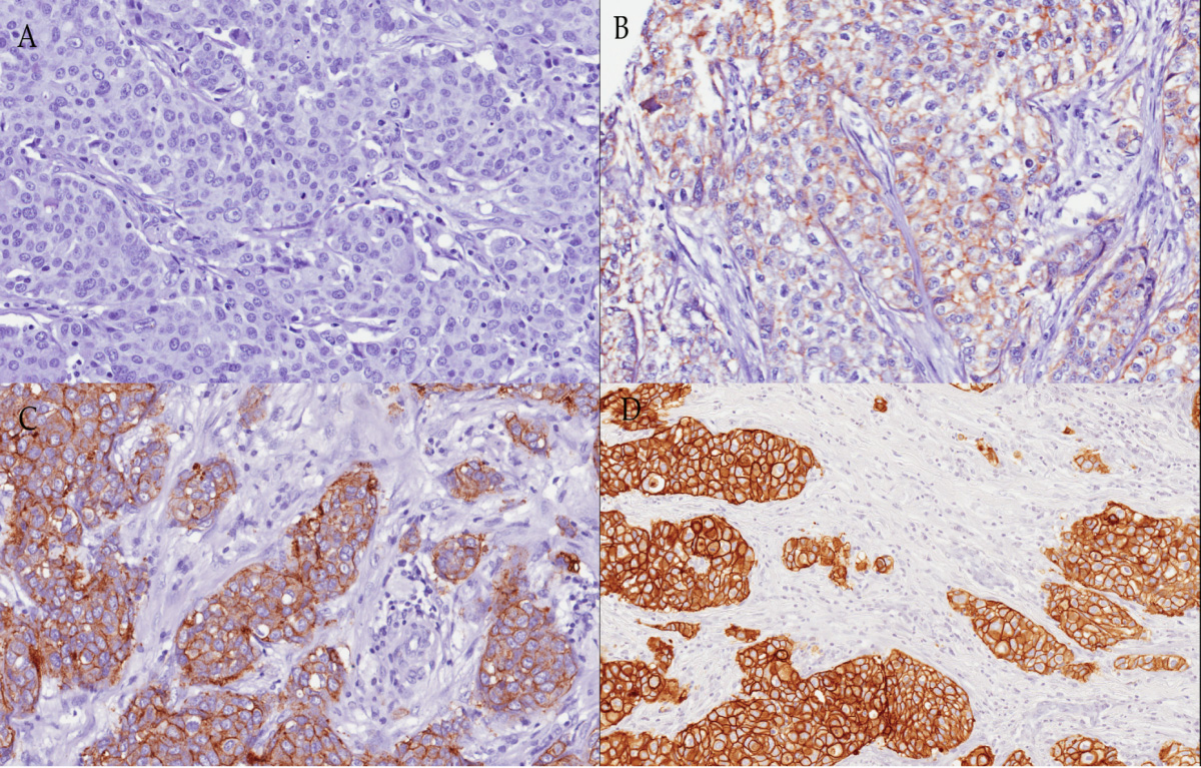
**Immunohistochemical staining of EGFR in triple negative breast cancers**. **(A) **Negative EGFR expression **(B) **1+ EGFR expression **(C) **2+ EGFR expression **(D) **3+ EGFR expression.

Genomic DNA was extracted from 70 cases of triple negative breast cancers. Successful PCR and good sequencing data were obtained in all samples except 7 samples, 6 samples and 14 samples that failed to amplify for Exon 18, 21 and 20, respectively. EGFR immunohistochemical staining results are shown in Figure 1.

### Detection of EGFR mutations

**Figure 2:**
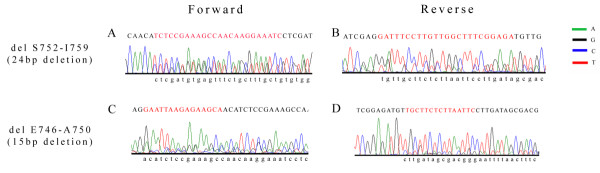
**Exon 19 deletions encountered in triple negative breast cancers**. Diagrams **A **to **D **show nucleotide sequences of EGFR gene in triple negative breast tumour specimens with heterozygous in-frame deletions within Exon 19 tyrosine kinase domain (double peaks). Tracings in both **(A, C) **sense and **(B, D) **antisense directions. The wildtype sequence is shown in capital letters, while the deleted mutant sequence is in lowercase letters. Deleted sequence is highlighted in red capital letters. (A, B) 24 bp deleted region of EGFR gene leading to removal of SPKANKEI at codons 752 to 759. (C, D) 15 bp deleted region of EGFR resulting in deletion of ELREA at codons 746 to 750.

**Figure 3:**
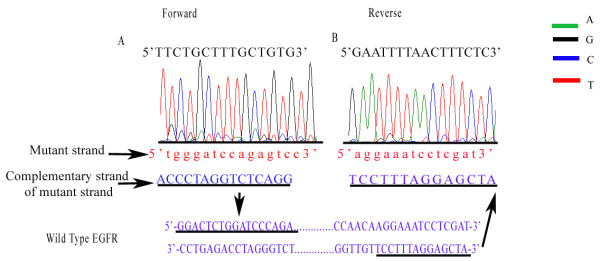
**Exchange of positions of double strands in EGFR exon 19, accompanied by gene inversions**. Diagrams **A **and **B **show nucleotide sequences of EGFR exon 19 gene in triple negative breast tumour specimens with heterozygous gene inversions of the complementary strand (double peaks). Tracings in both (A) sense and (B) antisense directions. The wild type sequence is shown in black capital letters, while the mutant sequence is in red lowercase letters. The complementary sequence of the mutant strand corresponds exactly to the wild type sequence and the orientation is reversed. Note only a segment of the sequencing diagrams (reading sequence towards the end) of the forward and reverse sequence is shown to demonstrate that the whole portion of exon 19 gene is inverted with exchange of the complementary strands.

Several different mutations located in different exons were found in triple negative breast cancer (Table 4). Overall, 8 of 70 cases (11.4%) of triple negative breast cancers showed heterozygous exon 19 deletions or exon 21 missense substitution mutations. Notably, 4 of 70 samples (5.8%) had in-frame deletions in exon 19, where 2 samples (2.9%) demonstrated 24 bp nucleotide deletions at mRNA coding sequence position 2254 to 2277, resulting in removal of eight amino acids Serine-Proline-Lysine-Alanine-Asparagine-Lysine-Glutamic acid-Isoleucine (SPKANKEI) at codons 752 to 759 (del S752 to I759) (Figure 2A, B) and the other two samples (2.9%) had a 15 bp nucleotide deletion at mRNA coding sequence positions 2236 to 2250, with the deletion of five amino acids Glucine-Leucine-Arginine-Glucine-Alanine (ELREA) from codons 746 to 750 (del E746 to A750) (Figure 2C, D). We also observed an exchange of positions of the double strands in the EGFR exon 19 gene, which was accompanied by gene inversion in one sample (Figure 3). Corresponding normal benign breast tissue of all the above cancers harboring mutations did not disclose any abnormalities.

**Table 4:** Summary of EGFR mutations detected in primary tumours of triple negative breast cancers (*n *= 8)

Mutations	Number of samples (%)
*Exon 19*	
del E746 to A750 (15 bp deletion)	2/70 (2.9%)
del S752 to I759 (24 bp deletion)	2/70 (2.9%)
inversion of complementary strand	1/70 (1.5%)
	
*Exon 21*	
L858R	1/70 (1.5%)
T847I	2/70 (2.9%)
**Total**	**8/70 (11.4%)**
	
**Single nucleotide polymorphisms**	
*Exon 18*	
T725T	3/70 (4.3%)
	
*Exon 20*	
Q787Q	6/70 (8.6%)

**Figure 4:**
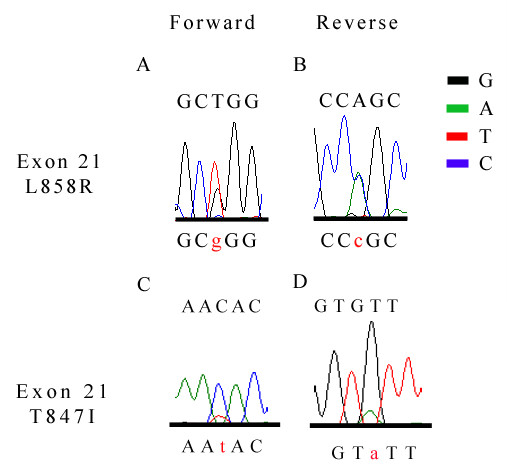
**Exon 21 missense mutations seen in triple negative breast cancers**. **(A, B) **Diagrams show substitution of T to G at mRNA coding nucleotide sequence 2573; leucine to arginine amino acid change at codon 858 (L858R). **(C, D) **Substitution of C to T at mRNA coding nucleotide sequence 2540, resulting in a threonine to isoleucin substitution at amino acid codon position 847. Tracings in both (A, C) sense and (B, D) antisense directions. The wild type sequence is shown in capital letters, while the missense nucleotide is highlighted in red lowercase letters.

Mutations detected in exon 21 of triple negative breast cancers included two different missense mutations: a T to G substitution at mRNA coding sequence position 2573 resulting in a Leucine to Arginine change at codon 858 (L858R) in 1.5% of the triple negative breast cancers (1 of 70 samples) (Figure 4). We also observed a missense substitution of a Threonine residue to an Isoleucine at amino acid codon position 847, resulting from a nucleotide substitution of C to T at mRNA coding sequence position 2540 (2 of 70 samples). Similarly, corresponding normal breast tissue of the above three mutated cancer samples showed no mutations, confirming their somatic nature.

Additionally, at exon 18, we found a single nucleotide polymorphism at mRNA coding sequence position 2175 (ACG to ACA) that led to no amino acid changes at codon 725 (Threonine to Threonine) in 4.3% (3 of 70 samples) of the triple negative breast cancers (T725, NCBI Single Nucleotide Polymorphism database, rs55959834). Also, G to A single nucleotide polymorphism at codon 787 (Glutamine to Glutamine) of exon 20 was found in 6 of the 70 (8.6%) triple negative breast cancers samples (Q787, NCBI Single Nucleotide Polymorphism database, rs1050171). Corresponding normal breast tissues revealed the same polymorphisms as observed in the invasive cancers.

Our study also revealed that triple negative breast tumours harboring EGFR mutations consisted of both EGFR protein positive and negative-stained cases by immunohistochemistry (Table 5).

**Table 5:** EGFR immunostaining scores for 8 samples with EGFR mutations

Sample no.	EGFR IHC score	EGFR mutation
1	0	T847I (in frame deletion mutation)
2	0	T847I (in frame deletion mutation)
3	0	Inversion of complementary strand of exon 19
4	3+	Del S752 to I759 (In frame deletion mutation)
5	2+	L858R (Missense mutation)
6	1+	Del E746 to A750 (In frame deletion mutation)
7	1+	Del E746 to A750 (In frame deletion mutation)
8	0	Del S752 to I759 (In frame deletion mutation)

IHC, immunohistochemical.

## Discussion

In this study, we report the presence of EGFR mutations, notably exon 19 deletions and exon 21 missense (L858R) mutations, in 11.8% of triple negative breast cancers evaluated. All samples showed heterozygous deletions, suggesting they are likely dominant and play a role in tumourigenesis [13]. EGFR mutations have been found to occur in 13% to 64% of all non-small cell lung cancers [27] and exon 19 deletions and L858R mutations account for >80% of all EGFR mutations detected in NSCLC [27]. More significantly, there is a plethora of studies demonstrating that patients with these exon 19 deletions and L858R mutations respond very well to EGFR inhibitors in NSCLC, as summarized in several reviews [27-29]. Exon 19 deletions and L858R mutations cluster around the ATP-binding pocket of the kinase domain and such mutations confer ligand-independent activation and increased activation duration compared to wild-type receptors [13,14,30,31]. *In vitro *studies showed that exon 19 deleted mutants and L858R mutant receptors appear to be more sensitive to gefitinib inhibition as compared to wild type receptors [13,14]. This could explain why NSCLC patients harboring such mutations respond better to EGFR tyrosine kinase inhibitors (TKIs) than patients without such mutations. Patients with exon 19 harboring deletions were found to have longer survival following treatment with gefitinib or erlotinib compared with those having L858R mutations in NSCLC [32,33]; however, Marks *et al. *reported no difference in survival between exon 19 deletions and L858R mutations in the absence of EGFR targeted therapy [34].

Several variant types of exon 19 deletions have been reported in NSCLC where the majority (65% to 75%) have EGFR exon 19 deletions of the 15 bp type (ELREA) and only a minority (1 to 1.5%) have 24 bp exon 19 deletions (SPKANKEI) [35]. Conversely, in our triple negative breast cancers, we discovered that both exon 19 type deletions (del E746 to A750 and del S752 to I759) are seen at similar frequencies. Correlative studies where exon 19 deletions were found to predict good response to EGFR TKIs mainly include the 15 bp deletion type, while 24 bp deletion types were rarely included (probably due to its low frequency) or at best, analysed as a group with the 15 bp deletion mutant. To our best knowledge, there appear to be no reports addressing differing sensitivity of different EGFR exon 19 deletions to gefitinib or erlotinib therapy. Future studies can be done to determine whether there is a variation in sensitivity to EGFR TKIs between the 15 bp deletion type and 24 bp deletion type.

Bhargava *et al. *reported no EGFR mutations in 11 EGFR-amplified sporadic breast tumours examined, out of which eight tumours were triple negative [36]. In a group of 47 metaplastic breast carcinomas which belonged to a subset of basal-like breast cancers, no EGFR tyrosine kinase mutations were identified as well; however, the actual ER, PR and cerbB2 status of these tumours analysed were not explicitly clarified [21]. Generali *et al. *also reported no EGFR mutations in 42 sporadic breast tumours (no selection for triple negative breast tumours) [37]. In a study of 58 triple negative breast tumours from Japanese patients, using Taqman genotyping assays against 14 known EGFR mutations including those for exon 19 deletions and L858R missense, EGFR mutations were also not found [38]. However, Weber *et al. *detected a higher rate of EGFR missense mutations in BRCA1/2 positive tumours (45.8%) compared with sporadic breast cancers (14.6%) [39]. BRCA1-linked breast tumours typically exhibit triple negative expression (ER-/PR-/Her2-) [12], express basal cytokeratins 5 and 6, and EGFR [40-42] and show similar histopathological features to basal-like/triple negative breast cancers [42]. However, EGFR missense mutations identified in the BRCA1/2 tumours were different from those encountered in our triple negative breast cancer study, possibly due to different patient selection where the BRCA1/2 linkage of our study cohort is unknown. The apparent differing findings of the above reports with ours could suggest heterogenous EGFR genomic instability in different breast cancer groups and raises the need for selection of specific breast cancers for EGFR mutation analysis. Different patient ethnicity and backgrounds may also be a contributing factor to the contradictory results seen in our predominantly Chinese cohort with those of the Japanese study group.

The development of anti-EGFR directed therapy in triple negative breast cancer has been supported by the availability of some preclinical data. *In vitro *studies on effects of EGFR inhibition in triple negative breast cancer cell lines revealed that gefitinib inhibited EGFR phosphorylation, which led to reduced signaling by the mitogen activated protein kinase (MAPK) and Akt pathway and causing cell cycle arrest at G1 phase [43]. In addition, gefitinib enhanced chemotherapeutic response to both carboplatin and docetaxel in these cells [43]. In a Phase II trial of erlotinib in patients with advanced breast cancer, 2 of 69 patients had partial responses, one of which had triple-negative histology [44]. Clinical studies are currently underway to evaluate the efficacy of EGFR tyrosine kinase inhibitors in patients with triple negative breast cancers.

As observed from our study, there is incongruity between EGFR positive immunostaining and the presence of EGFR mutations. It appears that positive EGFR protein expression does not predict the presence of mutations in triple negative breast tumours; conversely, mutations have been found in negative EGFR immunostained breast tumours. Monoclonal antibodies recognize extracellular epitopes of the EGFR and mutations in the EGFR gene could confer structural conformational changes that render them unrecognizable by the antibodies; besides, there are also possibilities of mutations in other exons of the EGFR that were not probed in our study. Pinter *et al. *have described that EGFR mutations in lung adenocarcinomas are not consistently accompanied by EGFR protein positivity by standard immunohistochemistry [45]. They reported that some of the EGFR mutant patients with major clinical response to gefitinib or erlotinib showed negative EGFR immunostaining [45]. Other studies have also noted such disagreement between EGFR immunoexpression and presence of mutations detected via molecular diagnostic methods [37,46]. These results suggest the importance of molecular diagnostic methods to identify EGFR mutations and subsequently those who will benefit from EGFR inhibitor therapy. Specific antibodies that can detect EGFR mutants have been developed by Brevet *et al.*, and could be advantageous in early identification of candidates for EGFR mutational analysis via molecular methods in the near future [35].

The relatively small sample size used in this study renders it difficult to make significant statistical judgments. Nevertheless, the identification of important EGFR mutations in our randomly selected cases from the larger series of triple negative breast cancers highlights the need to understand the frequency and type of EGFR mutations in a larger cohort, in order to promote a deeper understanding of the prospective utility of screening for EGFR mutations in relation to the therapeutic use of EGFR tyrosine kinase inhibitors for triple negative breast cancer in a clinical trial setting.

## Conclusions

This study demonstrated existing EGFR mutations in a relatively small study size of 70 triple negative breast cancers in a Singapore population (8 of 70 samples). Mutations observed included EGFR exon 19 deletions (4 of 70 samples ) and EGFR exon 21 substitutions (3 of 70 samples), both of which are commonly found in NSCLC and are good predictors of sensitivity to tyrosine kinase inhibition therapy. This advocates the promising application of gefitinib or erlotinib therapy in triple negative breast cancer, where the need to find new tailored treatment is critical. These findings also encourage larger scale prospective trials to evaluate the need for EGFR mutation screening for anti-EGFR treatment in triple negative breast cancers. It is also apparent that EGFR positivity by standard immunohistochemistry is not necessarily accompanied by EGFR mutations, suggesting that molecular diagnostic methods appear to be more important for selection of potential prospective patients with triple negative breast cancers who may benefit from EGFR inhibitor therapy.

## Abbreviations

Bp: base pairs; cerbB2: epidermal growth factor receptor 2; DNA: deoxyribonucleic acid; EGFR: epidermal growth factor receptor; ELREA: amino acids Glucine-Leucine-Arginine-Glucine-Alanine; ER: estrogen receptor; FFPE: formalin-fixed and paraffin-embedded; IDC: invasive ductal carcinoma; ILC: invasive lobular carcinoma; MAPK: mitogen activated protein kinase; NSCLC: non-small cell lung cancer; PCR: polymerase chain reaction; PARP: polyADP-ribose polymerase; PR: progesterone receptor; SPKANKEI: amino acids Serine-Proline-Lysine-Alanine-Asparagine-Lysine-Glutamic acid-Isoleucine; TKIs: tyrosine kinase inhibitors.

## Competing interests

The authors declare that they have no competing interests.

## Authors' contributions

YHFT participated in the design of the study, DNA extraction, PCR, analysis of DNA mutations and writing of the manuscript. WJT participated in DNA extraction, PCR, analysis of DNA mutations and writing of the manuscript. AAT participated in the design of the study, analysis of EGFR immunoscoring, and writing of the manuscript. PYC participated in EGFR immunostaining, DNA extraction and writing of the manuscript. GMKT, BHB, GWCY and NSW contributed to the scientific content and participated in writing the manuscript. PHT conceived the study, designed and coordinated all experiments and was involved in writing of the manuscript. All authors read and approved the final manuscript.
